# Highlights from the 2018 WIN Symposium, 25–26 June 2018, Paris: designing the future of precision oncology

**DOI:** 10.3332/ecancer.2018.871

**Published:** 2018-09-12

**Authors:** Will Davies

**Affiliations:** ecancer Global Foundation, 13 King Square Avenue, Bristol BS2 8HU, UK

**Keywords:** personalised medicine, genomics, biomarkers, targeted therapy, sequencing, precision oncology, trial design

## Abstract

The Worldwide Innovative Networking (WIN) Symposium is the annual gathering of WIN consortium members from across the globe, representing academic institutions, pharmaceutical partners, technology companies and charitable organisations, to discuss ongoing research and the latest developments in precision oncology. The symposium held in Paris, France on 25–26 June was structured into five plenary sessions, two open forums and poster presentations.

This year marked the 10th anniversary of the consortium, and the programme anchored itself with retrospectives of recent breakthroughs in personalised medicine, programme sessions reviewing the iterative design of trials in precision oncology at present, and the future of implementing personalised medicine initiatives in US and EU healthcare systems for the maximum patient benefit.

This year was also marked by the absence of Dr John Mendelsohn, who has stepped down as chairman, and co-founder Professor Tomas Tursz, who passed away in April this year. The latter was given a brief memorial session at the conclusion of the symposium.

Proceedings began with Richard Schilsky (American Society Of Clinical Oncology, USA), Chairman of the Worldwide Innovative Networking (WIN) Consortium, offering opening remarks and looking at the growing experience and reputation of the consortium. Recapping the global participation and mission of the network, as outlined above, he led into a brief video from chairman emeritus John Mendelsohn, which reiterated the importance of RNA targeting and pursuing unexpected changes in cell dynamics to deliver the next generation of cancer care. This led to a summary of the WINTHER trial [1] which matched tumour and normal tissue biopsies to deliver precision treatment based on RNA expression, and how lessons from that trial are being carried forward to the SPRING family of trials. Most notably, in informing the selection of a triplet therapy combination in place of monotherapy, and the development of an integrated DNA/RNA-based algorithm called Simplified Interventional Mapping System (SIMS). Further WIN platforms, the BOOSTER biomarker validation platform and the MERCURY drug development schema, were introduced ahead of their later discussion, and Dr Schilsky brought his introduction to an end with congratulating WIN members for all the consortium had achieved, had become and had set its course on pursuing.

Razelle Kurzrock, (UC San Diego Moores Cancer Center, San Diego, USA), chair of the Clinical Trials Committee, then took to the podium for a more in-depth discussion of the WINTHER trial starting with patient stratification; patients who had their normal organ tissue and tumour tissue RNA assayed through Agilent dual colour screening then had their treatment selected based on WINTHER algorithm indications for the best transcriptome-based therapy, or if next-generation sequencing did not pick up an actionable mutation to inform DNA-led therapy selection. In the latter case, choices were advised upon by the Clinical Management Committee in a weekly teleconference, with final decisions made by the treating physician. Dr Kurzrock went on to detail the patient cohort, by disease and geographical distribution, and how 303 consented patients were eventually selected down due to biopsy quality and treatment options to 69 patients in the DNA-led arm, and 38 in the RNA-led arm. Ultimately, the primary objective of the WINTHER study of achieving 50% progression-free survival (PFS) 2/PFS1 > 1.5 in the DNA arm, and 40% in the RNA arm was not met, and Dr Kurzrock considered whether the heavy pre-treatment of some patients impacted findings, and if the endpoints were too ambitious, compared to the 20%/23% rates achieved. She also noted that clinical benefit rates for the study, with stable disease, partial response and complete response data detailed by arm, was 26% for all patients. Another aspect Dr Kurzrock emphasised was the need for post-hoc analysis to account for the degree of matching to treatment, as patients were not always treated with a directly matched therapy. As such, those who received a highly matched treatment saw a greater PFS and overall survival (OS) benefit than those with a low match score.

Richard Schilsky returned to the podium to give the keynote lecture of the first presidential session, entitled ‘the challenges of building real cooperation to implement precision oncology programs: a long and winding road’. He reported on the TAPUR family of clinical trials aimed at identifying potential off-label indications for approved drugs. The first was an American Society of Clinical Oncology (ASCO) sponsored trial in the US, reported here, and subsequent international versions of this trial schema were highlighted, including the DRUP study in the Netherlands and a Canadian trial, CAPTUR. Dr Schilsky introduced a global trial of similar design being assembled by the WIN consortium, with participating members on four continents. With key elements in the trial design, endpoints and statistical analysis kept consistent across this family of trials, Dr Schilsky discussed plans for a shared data platform to combine translational genomic results from around the world.

Global solutions for global health problems were a key theme of Professor Stephen Hahn’s talk, which set out his experience and ambition in Making Cancer History all together. He opened with a summary of challenges facing the development and implementation of precision medicine in oncology, highlighting the need to make truly significant improvements in outcomes, developing optimal combinations of therapies without intolerable toxicity and gaining more complete insights into mechanisms of resistance and sensitivity. To these, he offered a recap of programs working to overcome each challenge, including the WIN SPRING triplet combination trial, which was discussed further later in the symposium, the MD Anderson Moonshot platforms and the key role of international initiatives such as WIN in opening access to information for all stakeholders and the global oncology community. Professor Hahn also discussed the IMPACT study, presented by Professor Apostolia-Maria Tsimberidou (MD Anderson Cancer Center, Houston, USA) at ASCO 2018 [2] and the subject of her own presentation on Tuesday afternoon, which matched patients with rare tumours who had exhausted their standard treatment options to therapies based on molecular analysis of their tumours to determine any targetable mutations. He reiterated the improved OS rates at 3 and 10 years compared to non-matched patients (15% *versus* 7%, 6% *versus* 1%), and the prognostic implications of PI3K/Akt/mTOR mutations. Professor Hahn concluded his session with a look at the ‘Dream List’ of personalised oncology, similar to that discussed by John Mendelsohn previously) [3], and how the goals of integrating Big Data and biomarkers beyond genomics are becoming realised elements of care, through WIN initiatives such as the WINTHER and SPRING trials.

Next, Dr James Doroshow (National Cancer Institute, NIH, Bethesda, USA) spoke about the perspectives of the US National Cancer Institute (NCI) on taking precision oncology from theory and research to bedside practice. He raised the same challenges facing precision oncology in practice as Professor Hahn, noting inadequate preclinical modelling for precision agents, nonstandardised data and tissue collection, and how NCI initiatives can address these issues on a national level. In the case of modelling, he discussed the NCI patient-derived models repository, which includes live imaging data, mouse models, organoids, and hundreds of patient-derived xenografts with a wealth of publicly accessible data. Access to these models feeds back into the network of trials and participants supporting the NCIs early-phase clinical trials. Beyond mouse models, he also discussed the utility of canine models to evaluate targeted and immunomodulatory therapies in comparable diseases. To address the lack of validated prognostic indicators that may predict response to immunotherapy, Dr Doroshow outlined the short long-term goals of a Cancer Moonshot Initiative in identifying translational biomarkers; the opening of four Cancer Immune Monitoring and Analysis Centres (CIMACs) and a Cancer Immunology Data Commons (CDIC), to enhance, centralise and open up data from academia, industry and other partners. In closing, he considered lessons learned from the NCI-MATCH trial for further efforts in matching therapies, and the future of precision oncology in a clinical landscape which continues to support sharing sequencing data.

Dr Josep Tabernero (Vall d’Hebron Institute of Oncology, Barcelona, Spain) spoke next about the European Society for Medical Oncology (ESMO) 2020 vision of delivering precision oncology to patients, built on integrated and sustainable approaches to cancer care, and providing specialised education. Key pillars to the integrated and sustainable cancer care he outlined were the provision of essential medicines, on which ESMO has issued recommendations for policymakers to avoid any shortages, and the development of new, innovative drugs. The geography and economics of delivering new and essential cancer treatments weigh unfavourably for many patients, and Dr Tabernero discussed the tools available for payers and policymakers to inform best care recommendations, including a geographically-adapted value-based reimbursement model. Dr Tabernero also discussed the involvement of ESMO in international discussion with policymakers for trials to address rare cancers and support the development of precision medicine, with a special emphasis on data protection. He cited examples of shifting trial designs, including umbrella and basket studies of targeted agents, and European Medicines Agency workshops on single-arm trials. Looking to the future, Dr Tabernero discussed how immunohistochemical and checkpoint biomarkers could be the tip of the iceberg in targets for precision oncology, with exome sequencing, prediction of neoantigens and hypermutation as potential developments. Beyond that, combined therapies and adaptive trial designs which complement the design of WINTHER, such as the FOCUS4 trial, the MoTriColor program and a proposed Basket of Basket study, promise to push the boundaries of how cancer is understood and managed.

Further European plans for innovative research were discussed by Dr Cornelius Schmaltz (Directorate General for Research and Innovation at the European Commission, Brussels, Belgium), in his session on the future of EU framework programmes for research and innovation. The EU Horizon 2020 initiative issued over €900 m of grants to over 650 cancer research projects, supporting technology and infrastructure development, leadership and addressing societal challenges, with cancer as a global health priority. As the project comes to the end of its 6-year arc, the follow-up—Horizon Europe—has been proposed by the European Commission to fund €100 billion over 7 years to strengthen science in the EU, and Dr Schmaltz discussed how aspects of the programme will impact health care and oncology. He highlighted the links between the Health Cluster of Horizon Europe and core research goals in academia, innovation, building expertise and open science. With six main areas of intervention in health throughout life, environmental/social determinants, noncommunicable diseases, infectious diseases, digital solutions and health care systems, cancer care, prevention and burden will be essential parts of research in the project, and Dr Schmaltz called for research with societal relevance, ambition, clear direction and cross-disciplinary goals to tap into the network and funding opportunities Horizon Europe presents.

This handily led to the forum session, ‘The Key Issues of Worldwide Cooperation’, featuring Dr Tabernero, Dr Doroshow, Dr Schilsky, Dr Schmaltz, Dr George Wilding, Lisa Hutchinson and Professor Jean Charles Soria, to discuss topics including precision medicine beyond DNA mutation, alternatives to DNA and RNA markers, and how to embed immunotherapy within precision medicine programmes. Dr Schilsky opened with the current use of transcriptomics in assays and screening for guiding therapies, followed by panel discussion on the statement of terms in what is meant by personalised, precision or targeted therapy and the impact of tumour burden on selection and timing of precision oncology. On the topic of supporting precision medicine in clinical decisions for payers and in reimbursement scheme, delivering irrefutable evidence of better efficacy and cost was identified as the most effective means of convincing healthcare providers, and that such evidence needs gathering and validation. Funding decisions discussed earlier by Dr Schmaltz came back in an audience question, which highlighted that all trans-retinoic acid ability to induce complete remission in almost all aute promyelocytic leukaemia patients was a breakthrough from a small lab, while much of the EU funding was going towards larger institutions. In the debate of immunoncology in precision medicine, Dr Schilsky made the case for immune modulatory agents sparing healthy tissues as precision component over checkpoint inhibitors, with innate immunity precise by itself whereas unleashing immune cascades wherever checkpoints are detected is comparably blunt. The uniformity of data gathering, archival and recall within clinics, national health systems and international collaborations was also raised as a barrier to incorporating Big Data in trial and treatments. Funding precision medicine trials was also discussed, with Dr Tabernero leading the panel in discussions of which European agencies are active and working across national boundaries, the role of charities in funding such trials, and how international collaboration and consistency has changed towards large transformational trials, and what oncologists and agencies can do to access their work.

After the break, the conference resumed with its second session: ‘the next frontier of therapeutics in newly diagnosed patients: standard of care or modern biomarker-driven precision oncology?’

First, Dr Leroy Hood (Institute For Systems Biology, Seattle, USA) took to the podium to discuss the P4 initiatives of his Institute of Systems Biology [3], and tracking a cohort of 4,000 ‘Wellness’ patients to identify their individual health states and transitions to disease with regular blood biopsies to identify any circulating biomarkers of interest. Ongoing sequencing and surveillance of patients could also be leveraged to identify changes in the transcriptome to signpost responses to treatment or disease relapse, and Dr Hood set out his goals of a sea change in 21st-century medicine towards ongoing promotion and maintenance of wellness over responding only to disease once it has become symptomatic.

Dr Amir Onn (Chaim Sheba Medical Center, Ramat Gan, Israel) spoke next introducing the BOOSTER trial from the WIN consortium. The trial aims to build a bio-repository of samples from the blood, tumour tissue and surrounding tissue of lung cancer patients, so that new blood biomarkers for nonsmall cell lung cancer (NSCLC) can be identified and validated. Dr Onn outlined the global aetiology of the disease, and limited benefits of screening studies with attempts at early detection being misled by false positives, low patient participation and high cost. Here, he said that liquid biopsy-derived biomarkers could build a comprehensive panel for early detection of disease and monitoring its possible relapse. The BOOSTER trial is running across the globe, and aims to recruit 4,000 stage 1 NSCLC patients for liquid biopsy, curative surgery and follow-up sample collection after 3 months, and Dr Onn invited all attendees to participate where possible.

Professor Harvey Pass (New York University Langone Medical Center, New York, USA) spoke further about lung cancer, offering lessons from a long-term survey of stage 1 patients and their disease. He opened with a summary of the New York University Lung Cancer Thoracic Surgery Archives, which were assembled to prevent false positives in imaging and aid with matching historic specimens to new patients for more well-informed diagnosis and treatment. This came with cautions on the logistics and representative accuracy of the data gathered, and comparisons of which samples, microenvironments and platforms are on offer. His final messages of the need for openness in archives led into a reiteration on the utility of the BOOSTER trial.

The second plenary session continued with Dr Ellen Sigal (Friends of Cancer Research, Washington DC, USA) presenting on behalf of patients and patient advocates, about patients driving progress. She opened with a brief history of the evolving voice of patients and advocates in their care, from passive observers or subjects to speaking up, claiming a voice through activism and awareness to being a part of policy and practice. In drug development, she pointed to the importance of patient perspectives being the human side to an otherwise molecular process. After touching on the Biden Moonshot initiative, Dr Sigal broached patient access to breakthrough therapies, which has just undergone a legal change permitting earlier access to trial therapies: Why, she asked, should patients have to wait years for drugs that show significant clinical activity in early development? With drugs that receive the breakthrough designation being approved months’ ahead others, she describes the allocation as having changed Federal Drugs Administration and NCI culture. A recent example is made of the LUNG-MAP Master Protocol, which has paired 1634 NSCLC patients to genomically matched novel therapies, with arms dynamically open and independent of others. With this trial evolving to accommodate new patients, therapies and genomic information, Dr Sigal emphasised that the patient experiences in trials and treatments must be the core outcome, and that research is in service to them.

The last speaker in the session was Professor Laura Esserman (UCSF Carol Franc Buck Breast Care Center, San Francisco, USA), discussing the past, present and future of precision oncology in breast cancer care, from the ‘One size fits all’ approaches of the 19th century, the introduction of radiation and refining surgical techniques through the 20th century, up to recent breakthroughs in screening, hormonal therapy and disease stratification. Professor Esserman emphasised the drive from patients for better research and better care, and the evolving predictive capabilities of clinicians to screen, prevent and understand the biological mechanisms of the many varied diseases within Breast Cancer. In terms of prognostic profiles, the question remains regarding which patients are at most risk of systemic recurrence. Here, Professor Esserman described a paradigm shift in understanding the variable progression rates and indications for different women, with those who show a trend of slow progression having the most benefit of early detection programmes, and those with indolent lesions or a rapid progression of disease having a limited benefit for early detection, benefiting instead from systemic therapy or best care as atypical cells eventually become cancerous. Within those women with indolent disease, a cohort exists with what Professor Esserman terms ‘ultra-low’ risk patients, whose disease can be stratified with a 70 gene panel [19]. She described the design of the WISDOM trial, currently recruiting patients, in which those who agree to randomisation will receive either routine annual screening or risk-based, adaptive screening to determine the safety, acceptance and preventative health value of personalised screening. As for prediction, Professor Esserman went on to address determining the optimal treatments for systemic disease and the significance of surrogate endpoints: taking a cue from the development of treatments for chronic myelocytic leukaemia (CML), she considered how testing new agents in patients with metastatic disease may not reveal the true potential of novel therapies, looking at the difference between drug responses in the accelerated and blast phases. Furthermore, the iSPY2 trial is testing drugs earlier in diseases development, with multiple drugs assessed in the neoadjuvant setting and a primary endpoint of partial clinical response (pCR) on ten biomarkers, so as to rapidly determine what therapies have the best chance to reduce dying of breast cancer. From a group of 746 patients across ten drug combination arms, Professor Esserman highlighted how pCR is an effective predictor of event-free survival and disease recurrence-free survival, and shared the results from the pembrolizumab trial arm showing a dramatic improvement over control, and high probability of success in phase III trials. Where the iSPY2 trial adapts therapy allocation within trials, the subsequent ISPY2+ is set to offer adaptive treatment choices per patient, validating surrogate markers based on pathway activity and increasing patients chance of pCR. Ultimately, Professor Esserman said that these trials are building a modern, personalised approach to breast cancer, with a more complete understanding of when to treat who has what type of disease with which therapy, increasing efficacy and reducing disease morbidity.

The panel session for the afternoon began with Dr Schilsky broaching the topic of breast cancer trials with Professor Esserman further, asking what could have been done to accelerate discovery beyond the 50-year timelines she identified in her opening slides. She countered that the surgical advances made were radical for their time, and that belief can sometimes trump evidence in the pursuit of science. As such, she holds regular meetings with a stakeholder panel to go over simulated trials results, so that plans to proceed can be drawn up now rather than in 5 years’ time when one of the simulated scenarios may come to bear. Now that a broad biological understanding is in place, Dr Hood added that data tracking and lifelong health management will expose early, actionable, personalised treatment options. In addressing an audience question of when and how underlying genomic changes can manifest as a primary lesion, Professor Esserman highlighted that the effect of hormone therapy on response biomarkers can point to patients in the low/ultra-low risk categories she described earlier, and then spare them from unnecessary treatment. On asking if the same could be said for lung cancer patients, which those at risk systemic disease should not be immediate candidates for surgery, Dr Pass countered that specimen-gathering limitations hold lung pathology back.

Another question on treating small primary lesions numbering only a few hundred cells with matched therapy was fielded first by Dr Hood, stating that the data gathered from many patients whose disease is managed as an *N* of 1 study will eventually indicate when to act in other patients, and which biomarkers of progression to be aware of Professor Esserman challenged that constant population screening was an expense beyond consideration, but that stratifying high-risk groups could do more to inform disease development and screening process, and that the lowest 15%–20% risk patients could continue without screening or revisiting. This struck some in the audience as a big risk for a large number of patients, to which Professor Esserman explained her position further; ‘I think we need to get out of the mindset that cancer detected at screening is a life saved. It is not’. With comparison to colon cancer screenings plans, and the likelihood of lung cancer screening only being offered to those already at a higher risk, Professor Esserman asserted that most women from her trial experience wanted tailored screening, and women and their families would be better served by reframing the conversation to change attitudes that not every cancer is a killer in need of immediate surgical excision.

When it comes to the incorporation of health record data, Dr Hood discussed integrating artificial intelligence (AI) to data to identify gaps in the record and draw actionable links within and between patients. He also described the need for a holistic approach to treatment and tracking of patients that ‘Cancer is not just tumour in an organ; it is all about their response… the same cancer in ten individuals will give you ten strikingly different outcomes’. Professor Esserman and Dr Onn picked up on the thread of record management, noting that many clinicians were still working with outdated tools that have only recently been modified to tracking smoking status.

The risk of disease resulting from prior treatment was highlighted as exposing resistant phenotypes as helping to better determine a sequence of treatment, as was the potential mediation of the microbiome. The semantics and trial design behind Breakthrough designation was raised, to which Professor Sigal insisted that clinicians respect the data as it emerges.

Dr Hood spoke about the power of embracing *N* of 1 studies to detect unexpected biomarkers or outliers that may inform broader disease management, which spurred discussion on the de-identification of data. Razelle Kurzrock commented that ‘I know everything about this patient, including their molecular profile’, and much of the panel share the opinion that, with such distinct data being generated, deidentification was perhaps no longer necessary.

When the panel was asked if they would, or could, adopt Dr Hood’s approach to *N* of 1, Dr Kurzrock highlighted the iPREDICT trial, in which each patient is treated as such. Dr Hood emphasised the need for generating as much data as possible to draw meaningful conclusions. With 4,000 patients in his health system currently, he reported an enthusiasm for data tracking and sharing, especially considering the lowering costs of assays in the near future. To this, the question was asked of when will those patients stop needing doctors if all their data are stored and analysed digitally?

Having discussed data deidentification, Dr Kurzrock considered that among small populations, randomisation approaches need flexibility, and some trials are not necessarily meaningful by the time they’ve concluded. The point is also raised for trials for the utility of randomisation when one arm is already clearly better—If you know which arm is better, randomisation is unethical. Dr Kurzrock commented that oncologists ‘should be striving for drugs so good that the randomisation is unethical’, and Professor Esserman reiterated the utility of pCR as an endpoint as of clinical significance, and significance for patients. Professor Sigal agreed that, with adaptive trial designs and signal finding in rare tumour types, this should be possible in lung cancer.

With this, the sessions for the first day closed.

The second day of the symposium opened with a session on disruptive concepts and trials that are shifting the paradigm of precision oncology, with Professor Yosef Yarden speaking first on antibody combinations to overcome resistance to kinase inhibitors.

From an initial consideration of the evolution of biological systems towards complex networks, and the concept of ‘centrality lethality’—targeting a central hub in a network is likely to do more damage than peripheral targets, Professor Yarden translated these ideas to lung cancer and the design of antibodies. He described how human epidermal growth factor receptor (HER) 2 and HER3 networks become upregulated when epidermal growth factor receptor (EGFR) alone is targeted, resulting in a tumour developing resistance to monotherapy, whereas a triplet therapy regimen to hit all the three surpassed any doublet in limiting tumour growth in mouse models. In the case of osimertinib, a tyrosine kinase inhibitor (TKI) which targets T790M NSCLC, he reported the emergence of a new resistant mutation to subvert even the latest generation of TKIs, though these tumours remain sensitive to triplet therapies. By combining the pro-apoptotic action of osimertinib with the induced senescence of the triplet antibody therapy, Professor Yarden reported a complete cure of mouse models. Professor Yarden highlighted that, while this is a significant result in mouse models, human disease management had better address the eventual toxicity of having four drugs. However, in experiments were any one antibody from the triplet was dropped (2xmAb + TKI), resistance and recurrence soon followed. With modulation of the drug-holiday period, an intermittent schedule of 1 week doublet + TKI treatment followed by 2-week break over the course of 4 months achieved near-total disease control. Similarly, early data from trials of mouse models receiving triple mAb therapy until the development of resistance, followed by doublet+TKI, are showing promising efficacy. Overall, Professor Yarden reported that the synergistic action of this type of treatment schedule is likely to have similar efficacy in other tumours, and similar combinations show promise for delivering effective cancer control in the future.

Dr Kurzrock took the topic of triple therapy and raised the stakes with design, goals and outcomes of the WIN-sponsored SPRING trial for metastatic NSCLC. She outlined how the WIN SIMS algorithm will leverage patient’s transcriptomic data to better understand tumours and the patients they are in, and their eligibility for tailored triplet cocktails to improve cure and avoid recurrence. Dr Kurzrock went on to break down the SIMS algorithm, which scores the transcriptomic activity of 24 RNA/DNA intervention points, and then sorts that score against a mutational database to identify actionable mutations. These scores expose a wide genetic variation between patients who have the ‘same’ disease, and also the genetic drift between healthy and tumour tissues from the same patient. The SPRING 01 study is the first in a planned series of trials, with subsequent investigations set to match tritherapy options for patients with different programmed death (PD)-1 expression, up to three lines of prior therapy and with different combinations of tritherapy including a C-met inhibitor.

Dr Jean-François Martini spoke next, raising appropriate concerns to the preceding presentations, addressing the challenges of combining targeted therapies in biomarker-driven precision oncology, from the perspective of a pharmaceutical company. He summarised the management of anaplastic lymphoma kinase (ALK)/ROS + NSCLC with crizotinib over the last 10 years, and went on to highlight the early inroads by lorlatinib, a third generation ALK inhibitor. As presented earlier by Dr Alice Shaw at American Association for Clinical Research (AACR) 2018 [4], lorlatinib compares favourably to other ALK inhibitors in treating G1202R mutated tumours, and has better brain penetration than crizotinib. He highlighted that, in patients who had previously received crizotinib as their only prior TKI treatment, lorlatinib induced responses regardless of ALK mutation status (72% *versus* 73%), and that those patients who had received multiple ALK TKIs, more responded if they did have an ALK rearrangement than not (61% overall response rate (ORR) *versus* 26%). Having summarised the lorlatinib data from AACR 2018, Dr Martini went on to summarise the findings from the JAVELIN trial presented by Dr Shaw at ASCO 2018 [5], which showed the combination of avelumab and lorlatinib for heavily pre-treated ALK + NSCLC patients was safe, with an ORR for 46%, but has not improve overall response of clinical response rates with data still maturing. Ultimately, the mechanisms of resistance to lorlatinib remains a mystery to solve, and Dr Martini stakes this as critical to developing the next generation of TKIs, alongside improved screening and refined liquid biopsy technology.

The last presentation in this session came from Dr Mary Beattie (Genentech, USA), discussing the MyPathway trial: a basket study of refractory, metastatic solid tumours in patients with a guiding mutation for their assignment to an off-label targeted agent. Dr Beattie discussed the benefits of basket trials, enabling studies in multiple tumours, rare cancers and detecting signals of activity, and the design and analytical considerations for MyPathway, with adaptive designs for tumours of differing origins and an assessment protocol independent of a common control group. Among the many baskets of the trial, she notes interesting rates of recruitment of patients with HER2 mutation/amplification from salivary, bladder and biliary cancers. Among these, she described ORR rates of up to 71% in salivary cancers treated with trastuzumab and pertuzumab. She highlighted the negative impact of KRAS mutation on ORR, especially among HER2+ colorectal cancer patients, and that these patients with be discounted from entering the trail going forward. From the BRAF + NSCLC basket, the results also showed data indicating a benefit of vemurafenib and cobimetinib for patients with V600 mutations. Co-mutations such of these will continue to inform trial recruitment and therapy selection as baskets open and close, and Dr Beattie considers how the trial design will be reflected in regulatory and reimbursement plans. She looks towards further, larger studies incorporating nonapproved drugs alongside off-label drugs, the development of companion diagnostics and the possibility for combining data from multiple sources.

Following a brief break, the symposium continued with its fourth session on new concepts and therapeutic avenues in precision oncology.

The keynote lecture from this session came from Dr Carl June (Perelman School of Medicine, Philadelphia, USA) on precision chimeric antigen receptor (CAR) *T* cells, a developing technique in immunotherapy which has seen breakthrough approvals (and prices) over the last year. He made the case in his opening comments that CAR *T* cells ought to be considered a process rather than a drug, and highlighted some key differences between currently approved cluster of differentiation (CD) 28 tisagenlecleucel and 4-1BB CAR T cells, including different vectors and persistence durations. Notably, the persistence of tisagenlecleucel is up to 7 years, to which Dr June describes pharmacokinetics of so-called memory CARs similar as part of the long-term success of this ‘living drug’. In the case of Emma Whitehead, a paediatric leukaemia patient whose recovery made the pages of the New York Times [6], he shared her results showing a deep remission induced over 3 weeks of treatment, with a complete response seen at over 5 years. From a Wright stain, Dr June also showed pictures of CAR T cells’ morphology and cerebrospinal fluid penetration. A key aspect of the CAR-T cells value and importance, he described, is their capacity of proliferate, dividing every 6 hours. However, some ALL tumours remain resistant through a shifting series of mutations leading to CD19 escape, and even transduction of tumour blast cells with CAR19. Dr June describes on the patient who experienced a loss-of-function mutation following the CAR-T induction in a Tet region. Looking towards future developments, Dr June noted the development of ‘Armoured CARs’ for solid tumours including prostate cancer, to evade resistance with less exhaustion, in a phase I trial currently open and accruing patients. He also pointed towards issues to address in centralised manufacturing on treatment availability, what may come of combined CAR therapies with vaccines, oncolytic viruses or checkpoint inhibitors and blood banking. The WIN symposium is probably the best place to ask such questions and get a very good answer.

The next session, immunotherapies turning cold tumours hot, was presented by Professor Jean Charles Soria. He opened the session by highlighting that while some checkpoint inhibitors had seen advances, immune exhaustion, suppression and absence still held many immunomodulatory therapies back. Oncolytic viruses offer one option to heat up immunologically cold tumours, if they can effectively prime new responses by inducing *T* cell recruitment and interferon expression to draw a full immune response to a tumour. Talimogene laherparepvec (T-VEC) approved in 2015, has had success, and shows promise for a combined efficacy with other immune agents, and Professor Soria discussed ongoing work with an engineered negative-strand RNA avian paramyxovirus [7] to induce a long-last antitumour immunity. When combined with PD-L1 blockade drugs, survival and response rates in a mouse model were dramatically increased. Professor Soria also raised antibody-drug conjugates (ADCs) as a means of delivering a precise strike to tumours, with the data from models of anti-ephrin A2 ADC [8], which also saw improved outcomes from added PD-L1 combinations. This is just one of 27 registered ADC + IO trials, according to Professor Soria, only one of which is currently in Phase III trials. A final consideration was of the role of CD40 as a master regulator of humoural and cellular immunity, and Prof Soria introduces a novel agent which bridges three CD40L subunits to mimic a natural ligand and spur the activation of host dendritic cells. He described the promise of this molecule in overcoming resistance and promoting immune maturation, and that it is now in a phase I trial as a combination with durvalumab for advanced solid tumours.

Dr Eric Rubin (Senior Vice President Global Clinical Development, Oncology Early Development, MSD) spoke on a similar biomarker theme in his presentation on precision oncology approaches for immunotherapy, opening with an adapted quote from George Box—No test is perfect, as recommended; some tests are useful’. From this, he led into a discussion of the PD-1/PD-L1/PD-L2 pathway, which has contributed to a broad change in biomarker and targeted therapy research over the last few years. The approval of companion diagnostics came ahead of the approval of first-line pembrolizumab for NSCLC itself, and Dr Rubin summarised results presented by Dr Leena Ghandi at AACR [9] showing a combined chemotherapy approach halved the risk of death for mNSCLC patients with PD-L1 < 1%. Pembrolizumab has also been approved in the US for patients with unresectable or metastatic, microsatellite instability-high (MSI-H) or mismatch repair-deficient (MMR) solid tumours, regardless of tumour site or histology, and Dr Rubin went on to consider whether PD-L1 inhibition could be attributed to the MSI-H phenotype, with data from ASCO 2015 [10] showing high rates of durable response in noncolorectal cancers with MMR deficiency. There have been several subsequent approvals of PD-L1 immunohistochemical assays, and PD-1 targeting drugs, leading to some efforts to establish a concordance [11], and Dr Rubin noted multiple assays are currently being developed to evaluate tumour mutational burden. Audience questions soon raised the volume of data being generated from multiple trials, and whether retrospective investigations with clonal diversity data from trials may refine predictive markers, or prognostic factors going forwards, and how much data would be enough? Here, Dr Rubin weighed whether a surplus of data offer more answers or obfuscate useful insights, and that it’s going to take close work with statisticians to find answers.

The last speaker in this session was Dr Giorgio Massimini (Merck KGaA, Germany) who asked if the maximum tolerated dose (MTD) and the efficacy of monotherapies were still valid as the best objectives in early oncology trials. Many trials incorporate pharmacodynamics and pharmacokinetic parameters to inform optimal Phase II dose beyond MTD, and Dr Massimini raises results from a dose modelling trial of tepotinib [12]— no MTD was reached up to 1,400 mg, while PK modelling showed an effective dose between 300 and 500 mg, with no dose limiting toxicities. As such, he describes, there is the need to establish better synthetic lethality, and consider how the timing of each therapy in a combination can be optimised. Dr Massimini considered alternatives currently on offer, including the use of basket and umbrella trials, and the development of the MERCURY drug platform as part of the WINTHER trial, as discussed earlier. The MERCURY program aims to develop combinations in a noncompetitive space, based on matched normal/tumour biopsy and RNA, algorithmic matching and compatible safety profiles. Dr Massimini rounded up his session with the conclusion that the value of MTD is rapidly diminishing, and that the future of early trials would be better served by a focus on target inhibition, safety and clinical outcome, effectively pooling the outcomes of phases I and II into flexible designs.

Some of these points came back for discussion in the following forum session, discussing the future of drug development in precision oncology. In terms of what pharmaceutical companies, academic centres, payers and consortia can provide to optimise deriving combinations, endpoints beside ORR were considered as a means to accelerate investigations, and that if the presence of a biomarker is not decisive in treatment response, to better track responses in patients who were lacked the biomarker and establish significance by its absence.

Referencing the data density discussed by Dr Hood, the potential volumes of data which could be gathered from any patient in a large cohort, the commonality of data definitions, electronic form structure, abbreviations and reporting mechanisms was highlighted by Dr Schilsky as a fundamental hurdle in logistics, and that an untrained AI would do little to resolve this Babel-like situation without core human curation. This may be started with pharmaceutical partners standardising their data tracking especially in the case of annotating combination therapies, and attendees were encouraged to take these conversations with them into future trial designs. Similarly, placing the onus on doctors to ‘just practice differently’ and go beyond what there is time, money and capacity to achieve in their clinic does little to address the underlying system, which will need time to change.

The impact of the microbiome on immunotherapy was described as still in its earliest stages, and the challenge of autoimmune conditions on cancer management with immunomodulatory remains unresolved. Dr June noted the existence of some anecdotal data impaired host defence and common epitopes on tumours as in dysbiosis, but these questions will take time to answer.

An audience question raised the historical role of academic centres in pioneering combination therapies, and asked what modern centres could do to push rational development of drugs without excessive trial costs. Dr Massimini noted the expiration of patents as offering inroads, and Dr Schilsky commented that several years of negotiation has now led to some NCI labs to conduct combination therapy research from multiple pharmaceutical providers. The panel was also asked how they saw conversations between regulatory and paying bodies changing in an increasingly competitive market place for biomarker-based therapy, to which the movement of the EMA to Amsterdam was raised as a significant bump in the road for predicting European approvals and reimbursement. Here again, Dr Schilsky recommended that the best course of action to open discussion was to keep making high-quality evidence.

Dr Kurzrock raised a final point that oncology, as a field of healthcare, is most averse to combining treatments, whereas treating a patient with multiple different medical issues in other fields of medicine is not a matter of much great concern. Therefore, ought not the combination of therapies based on a patient’s individual comorbidities be achievable? Dr Schilsky agreed that, while academic validation of every combination would be unfeasible, a proof of principle would make it easier to broach combining cancer therapies with treatments for other diseases, and ongoing data collection would be invaluable. Branching from meta-analyses to network meta-analyses was also offered as a statistical means of synthesising direct and indirect data.

The final plenary session, Challenges of Precision oncology, was opened by Professor Francesco de Lorenzo (European Cancer Patient Coalition (ECPC), Brussels, Belgium) in addressing the access of patients to precision oncology.

First, he raised the challenge of unequal availability of targeted drugs to cancer patients across Europe, and the need for flexible reimbursement to even the scales. Similarly, the cost of trials remains a barrier for participation. The strategy set out by the ECPC aims to support sustainable governance and empower its members and partners to participate in policy choices across the EU. The role of patients in their research is also a priority, and Professor de Lorenzo emphasised that personalised medicine is the future of cancer care, necessitating patient involvement. The first barrier he raised facing the widespread access to innovative medicines is that patient’s preferences are less of a priority to some than a statistical stratification. Beyond the underlying inequity of cost and affordability in differing countries, Professor de Lorenzo also identified the delay between innovations in research and national healthcare systems, and a need for a cultural shift from treating diseases to managing patients. In the case of trastuzumab reimbursement following its approval in EU members, the delay between approval and reimbursement varied from 0 to 2 months in many Western European countries, up to 12 years in Latvia. Similarly, a survey presented at ESMO in 2016 [13] highlighted a divide of 70% of patients in Western Europe being treated with innovative drugs compared to 10% of those in Eastern Europe have access to the latest recommended treatment in EU guidelines. Professor de Lorenzo called for the integration of quality of life and social economic benefit data to value-based judgements on drug availability, and questioned whether the current system of approvals for innovative medicines in emerging markets is itself counterproductive to increasing access to current treatments. He noted that a proposal for harmonised approaches in health technology assessment is currently passing through the European Parliament which would improve collaboration of regulatory bodies, speeding clinical assessment and streamlining drug access. Addressing barriers to biomarkers, Professor de Lorenzo raised that many countries do not reimburse biomarker assays, and others face delayed access to molecular test results. Current lobbying efforts from the ECPC focus on improving access and reducing these wait times, improving patient literacy around biomarkers, and harmonising the accompanying regulatory frameworks [14]. Professor de Lorenzo closed with a mention of the CanCon Joint Action [15], which the ECPC is now working to implement in EU member countries to ‘Improve access by updating obsolete procedures and eliminating waste to find funds for innovation. Patients should be at the centre of this process’.

Professor Apostolia Tsimberidou spoke next, moving from challenges to successes in implementing precision oncology with results from the IMPACT trials. These trials, presented at ASCO earlier this year, aimed to deliver on the core concepts of precision oncology, matching patients tumour molecular analysis to their treatments. She described the process of allocating patients to trials in which a matched drug would be available, with 54.4% of patients with a targetable alteration receiving a matched therapy, many of whom were heavily pre-treated. Out of all evaluable patients, Professor Tsimberidou reported that 16.2% of patients receiving matched therapy had an objective response compared to 5.4% nonmatched, and a 4% improvement in patients with stable disease beyond 6 months. Overall, this translated to a median OS benefit of 9.3 month *versus* 7.3 month, and Professor Tsimberidou emphasised the long plateau of survival, with 6% of patients from the matched therapy group alive at 10 years’ *versus* 1% from the nonmatched group. From multivariate analysis, she also described the significance of alterations in PI3K, AKT and MTOR pathways on risk, and the compounding effect of risk factors on limiting OS. Looking at the impact of nonmatched therapy as risk factor in further analysis, Professor Tsimberidou neatly summarised the findings of IMPACT1 as ‘Matched therapy is an independent factor associated with longer OS’. Facing the future, Professor Tsimberidou summarised the evolution in personalised cancer treatment since the inception of the IMPACT trial in 2007 to include immunotherapy and new biological understandings, and what still needs to be done to improve implementation and innovation in precision oncology. This is where the IMPACT2 trial aims to step in, matching patients with metastatic disease featuring a targetable alteration to clinical trials and commercially available targeted therapy. Enrolment in genetically matched trials is a core research priority at MD Anderson, with the Precision Oncology Decision Support database (as discussed by Dr Funda Meric-Bernstam at WIN 2016) [16], and Professor Tsimberidou highlighted the integration of clinical trial and NCI data into their workflow and information sharing platforms, available online through the MD Anderson website. She raises the NCI-MATCH and ASCO TAPUR trials, an exploratory study of nivolumab ± ipilumumab based on CD8 expression, and the ACTolog trial of endogenous CD8+ T cells as all recruiting patients, and encourages the audience to embrace the growing complexity in understanding biomarkers to uncover more effective drugs for more patients, faster.

Finally, Bernadette Toner gave the perspective of a news organisation on developments in personalised medicine in cancer care, and the ‘greatest hits’ of precision oncology. Top of the charts is trastuzumab, with its $7 bn revenue in 2017 and tremendous impact on preventing disease relapse and improving survival. Imatinib follows at $2 bn revenue, due to age and a newly available generic, but its impact on 5-year survival for CML patients from 30% to 90% cannot be understated. Working through approved EGFR and BRAF inhibitors, Toner described this data as admittedly drug focused, but that this is how the pharmaceutical companies and commenters are set up to gauge success. She went on to consider how to move to a more process-focussed model, as precision oncology tends away from large patient cohorts with common mutations towards rare markers and *n* = 1 studies. To reflect this, Toner said, a ‘success’ would be better defined in comprehensive strategies, producing optimal results for the most patients possible at the least overall cost. Value-based genomics and cost-effectiveness studies have so far been largely retrospective, and the cost of population-level screening to uncover the handful of patients with actionable mutations in rare cancers is not going to be manageable. For media perspectives and doctors alike, avoiding hype and placing gradual advances in their proper context can be made challenging by publicly visible advertisements and anecdotal evidence. Considering the attrition at every step of patient participation in clinical trials, from eligibility to biopsy to sequencing to matching criteria to drug access to response, anecdotal data is about where Toner estimates the evidence for precision oncology currently stands. Taking lessons from the SHIVA trial, and the trial design of IMPACT, NCI-MATCH and WINTHER studies, match rates and responses therein make it hard to accurately report on what a ‘good drug’ is, and means for patients. Oncologists, too, are faced with an increasingly complex field of treatment choices based in biomarkers, pre-treatment and ongoing approvals. Citing data from two recent surveys of doctors opinions [17, 18] on liquid biopsy and genome sequencing, Toner reported that the percentage of doctors who offer these new techniques is low, and their trust in its value is even lower. Evidence may come with time, and Toner encouraged the audience to continue their pursuit contributing to a better precision oncology model, making improvements at each step of trial participation, from time of testing for eligibility to new schema for drug matching and response evaluation. From the panel, Dr Schilsky considered that the greatest hits in precision oncology might be better considered as the discovery of biomarkers, such as oestrogen receptor and HER2 status rather than any one treatment, and Dr Kurzrock recommended that there would be value in encouraging patients to pursue sequencing earlier to counter the attitudes of some doctors in which it comes as a last step before hospice care. Dr Vladimir Lazar took this last point up later that when a patient comes to a doctor with the hope of survival, the barrier to prescription of drugs, assays and sequencing comes from the doctors, held back by either economics or education, and that is where change will come.

Before the closing session, awards were given to Dr Ruliao Liu and Dr Ferdinandos Skoulidis for their posters on ‘Dynamic monitoring HER2 amplification of circulating DNA in metastatic colorectal cancer patients treated with cetuximab’ and ‘STK11 mutations and PD-1 inhibitor resistance in KRAS-mutant lung adenocarcinoma, respectively’.

## Conclusion

The final session of the WIN symposium was a celebration of the life and achievements of Professor Thomas Tursz, former chairman of the consortium who passed away earlier this year. Professor Jean Charles Soria gave a few words on his life and accomplishments, reshaping Gustave Roussy to deliver world-leading clinical and supportive care, uplifting doctors and patient’s experiences, and cofounding the WIN consortium with Dr John Mendelsohn in 2008.
